# Leiomodin-3-deficient mice display nemaline myopathy with fast-myofiber atrophy

**DOI:** 10.1242/dmm.019430

**Published:** 2015-06-01

**Authors:** Lei Tian, Sheng Ding, Yun You, Tong-ruei Li, Yan Liu, Xiaohui Wu, Ling Sun, Tian Xu

**Affiliations:** ^1^ State Key Laboratory of Genetic Engineering and National Center for International Research of Development and Disease, Fudan-Yale Center for Biomedical Research, Innovation Center for International Cooperation of Genetics and Development, Institute of Developmental Biology and Molecular Medicine, School of Life Sciences, Fudan University, Shanghai 200433, China; ^2^ Howard Hughes Medical Institute, Department of Genetics, Yale University School of Medicine, New Haven, CT 06536, USA

**Keywords:** Leiomodin-3, Nemaline myopathy, Mouse, Fast-myofiber atrophy

## Abstract

Nemaline myopathy (NM) is one of the most common forms of congenital myopathy, and affects either fast myofibers, slow myofibers, or both. However, an animal model for congenital myopathy with fast-myofiber-specific atrophy is not available. Furthermore, mutations in the leiomodin-3 (*LMOD3*) gene have recently been identified in a group of individuals with NM. However, it is not clear how loss of LMOD3 leads to NM. Here, we report a mouse mutant in which the *piggyBac* (*PB*) transposon is inserted into the *Lmod3* gene and disrupts its expression. *Lmod3^PB/PB^* mice show severe muscle weakness and postnatal growth retardation. Electron microscopy and immunofluorescence studies of the mutant skeletal muscles revealed the presence of nemaline bodies, a hallmark of NM, and disorganized sarcomeric structures. Interestingly, *Lmod3* deficiency caused muscle atrophy specific to the fast fibers. Together, our results show that *Lmod3* is required in the fast fibers for sarcomere integrity, and this study offers the first NM mouse model with muscle atrophy that is specific to fast fibers. This model could be a valuable resource for interrogating myopathy pathogenesis and developing therapeutics for NM as well as other pathophysiological conditions with preferential atrophy of fast fibers, including cancer cachexia and sarcopenia.

## Abstract (precis)

**Highlighted Article:** A leiomodin-3 mouse mutant generated by insertion of the *piggyBac* transposon exhibits nemaline myopathy with fast-myofiber-specific atrophy.

## INTRODUCTION

Nemaline myopathy (NM) accounts for 17% of cases of congenital myopathy, with an estimated incidence of 1 in 50,000 individuals (Maggi et al., 2013; Wallgren-Pettersson, 1990). The characteristic features of NM include muscle weakness and the presence of rod-like structures (nemaline bodies) in skeletal muscle fibers (North et al., 1997). Based on age of onset and severity of symptoms, NM is clinically defined into six forms: severe congenital, Amish, intermediate congenital, typical congenital, childhood-onset and late-onset (Wallgren-Pettersson and Laing, 2000), although there is considerable overlap among the different forms. Accordingly, the clinical presentation of NM varies from lethality in early childhood to slow progression of milder defects in adults (Ryan et al., 2001). The type of myofiber that shows atrophy in individuals with NM also varies, from fast or slow fibers alone to both. Interestingly, preferential atrophy of fast fibers is also a common feature of other myopathy conditions, including cancer cachexia (Mendell and Engel, 1971) and sarcopenia (Lexell, 1995). However, an animal model for congenital myopathy with fast-myofiber-specific atrophy is not available.

Mutations in ten genes have been identified in individuals with NM (Agrawal et al., 2007; Donner et al., 2002; Gupta et al., 2013; Johnston et al., 2000; Laing et al., 1995; Nowak et al., 1999; Pelin et al., 1999; Ravenscroft et al., 2013; Sambuughin et al., 2010; Yuen et al., 2014). NM is inherited in an autosomal dominant or autosomal recessive manner depending on the mutation. The overall penetrance of NM is unknown, although cases of potential incomplete penetrance and sexual dimorphism have been reported (Agrawal et al., 2004; Nowak et al., 1999). Seven of the NM causative genes encode sarcomere thin filament proteins or regulators of their assembly, which suggests that disorganization of the thin filament could cause NM. Most recently, mutations in the gene encoding leiomodin-3 (LMOD3) on human chromosome 3, a newly identified protein localized to sarcomere thin filaments, have been detected in 21 NM patients from 14 families (Yuen et al., 2014). The mutant *LMOD3* alleles are inherited in an autosomal recessive manner. Consistently, knockdown of Lmod3 in zebrafish and *Xenopus* resulted in disorganization of thin filaments and muscle weakness (Nworu et al., 2015; Yuen et al., 2014). However, a mammalian *Lmod3* mutant model will be a valuable resource for interrogating the underlying pathogenesis of NM and for the development of therapeutics.

Here, we describe a mouse mutant in which the homologous *Lmod3* gene on mouse chromosome 6 is disrupted by a *piggyBac* (*PB*) transposon insertion. The *Lmod3^PB/PB^* mutant animals display severe muscle weakness in addition to growth retardation. Furthermore, *Lmod3^PB/PB^* muscle fibers display disorganization of sarcomere and the presence of NM bodies. Finally, *Lmod3* deficiency causes atrophy specifically in fast myofibers. Together, our study shows that *Lmod3*-deficient mice display NM and offers the first mouse model for congenital myopathy with fast-myofiber atrophy.

## RESULTS

### Generation of *Lmod3*-deficient mice

**Fig. 1.**
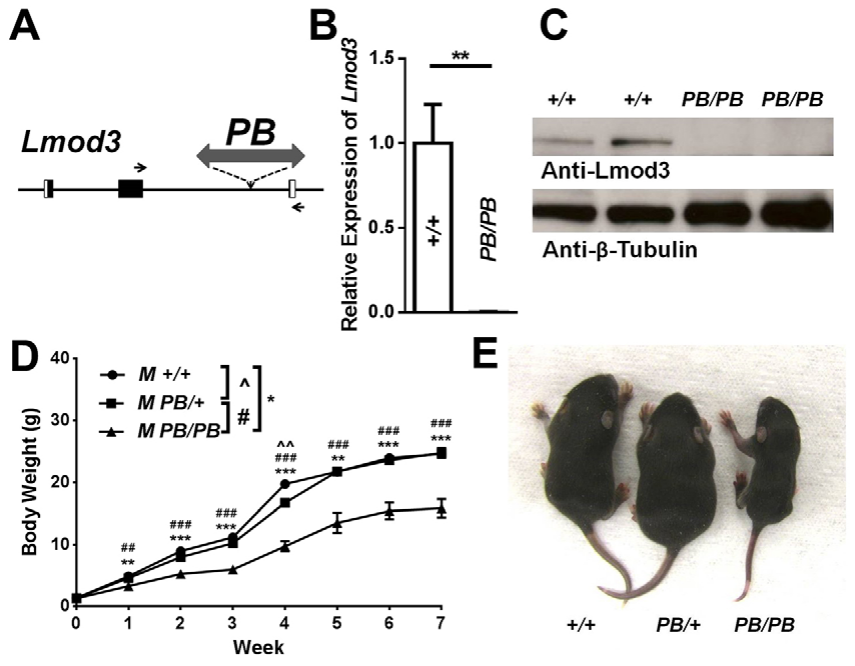
**Disruption of Lmod3 expression causes growth retardation in *Lmod3^PB/PB^* mice.** (A) Schematic representation of the genomic region of the *Lmod3* gene and the position of *PB* insertion. Black box: exon. White box: untranslated region (UTR). Arrows: primers for quantitative RT-PCR. (B) Quantitative RT-PCR analysis of *Lmod3* mRNA from TA muscles of 5-week-old mice with indicated genotypes. *n*=5∼7. (C) Western blotting of 5-week-old mouse TA muscles with antibodies against Lmod3 and β-tubulin (as loading control). (D) Growth curves of male *Lmod3^+/+^* mice (*n*=5), *Lmod3^PB/+^* mice (*n*=11) and *Lmod3^PB/PB^* mice (*n*=6). (E) A picture of three 1-week-old male littermates with genotype labeled. ^, # or * indicates *P*<0.05; ^^, ## or ** indicates *P*<0.01; ### or *** indicates *P*<0.001.

Using *PB*-mediated germline mutagenesis, we generated mouse mutants, each of which carries a single *PB* transposon insertion (Ding et al., 2005) (S.D., X.W. and T.X., unpublished data). In one mutant, *PB* is inserted into the second intron of the *Lmod3* gene (*Lmod3^PB^*) (Fig. 1A). Quantitative RT-PCR revealed that *Lmod3* mRNA expression is reduced to less than 1% of the wild-type control in homozygous mutants (Fig. 1B). Consistent with this, western blotting showed that Lmod3 protein is undetectable in *Lmod3^PB/PB^* mice (Fig. 1C). These results indicate that *Lmod3* expression is dramatically downregulated in *Lmod3^PB/PB^* mice. However, the expression of Lmod2, the other muscle-specifically expressed member of the Leiomodin gene family, was comparable between *Lmod3^PB/PB^* mice and wild-type controls (supplementary material Fig. S1).

**RESOURCE IMPACT**

**Background**

Nemaline myopathy (NM) is one of the most common forms of congenital myopathy, a group of muscle disorders present at birth. The characteristic features of NM include muscle weakness and the presence of rod-like structures (nemaline bodies) in skeletal muscle fibers. The type of myofiber showing atrophy in individuals with NM varies from fast or slow fibers alone to both. However, animal models of congenital myopathy with fast-myofiber-specific atrophy are not available. Recently, mutations in the gene encoding leiomodin-3 (LMOD3) have been detected in individuals with NM. However, the molecular mechanism via which loss of LMOD3 leads to NM is still unclear. Furthermore, preferential atrophy of fast fibers is also a common feature of other myopathy conditions, including cancer cachexia and other aging- or drug-induced myopathies. Currently no therapy is available to treat NM or other forms of fast-fiber atrophy.

**Results**

In the present study, the authors describe a mouse mutant in which the *piggyBac* (*PB*) transposon is inserted into the *Lmod3* gene to disrupt its expression. Mutant *Lmod3^PB/PB^* mice show severe muscle weakness and postnatal growth retardation. Moreover, the authors discovered disorganized sarcomeric structures and nemaline bodies in the skeletal muscles of the mutant mice. Interestingly, mutant animals exhibit atrophy specifically in fast myofibers, a unique clinical feature shown by only a subgroup of individuals with NM as well as other myopathy-affected individuals.

**Implications and future directions**

This study shows the first mouse mutant of NM that exhibits fast-myofiber-specific atrophy. The *Lmod3^PB/PB^* mouse is thus a unique mammalian model to study disease mechanisms and to dissect how *LMOD3* mutations can lead to NM. In addition, this model could prove helpful to develop therapeutics for both congenital and acquired myopathies that are specifically associated with fast-myofiber atrophy.

### *Lmod3^PB/PB^* mice show growth retardation and muscle weakness

*Lmod3^PB/PB^* mice were born at expected frequency with normal weight (Fig. 1D and supplementary material Fig. S2; data not shown). However, both sexes of *Lmod3^PB/PB^* homozygous, but not heterozygous, mice showed growth retardation as early as 1 week of age (Fig. 1D,E and supplementary material Fig. S2). By week 4, *Lmod3^PB/PB^* mice weighed 50% less than wild type (Fig. 1D and supplementary material Fig. S2), although all of them survived into adulthood.

**Fig. 2.**
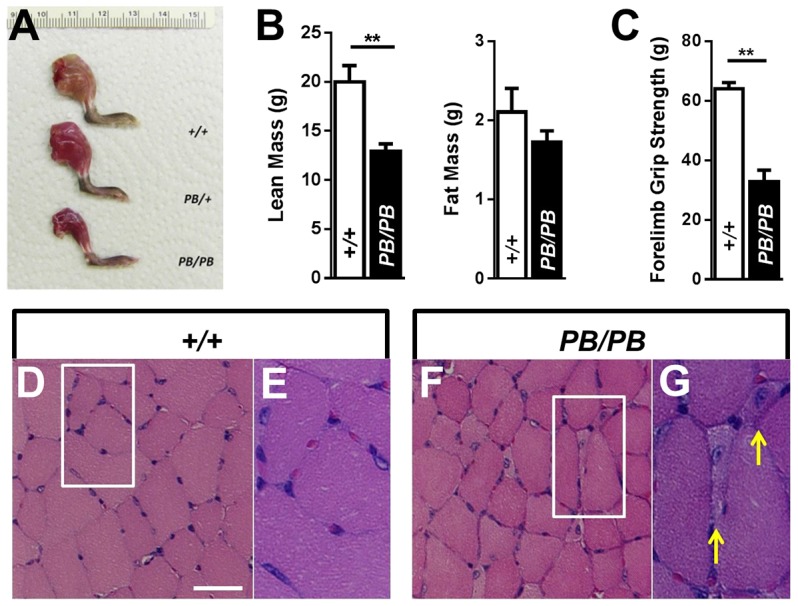
***Lmod3^PB/PB^* mice show muscle weakness due to atrophy of myofibers.** (A) A picture of unskinned hindlimbs of three 4-week-old male littermates with genotype labeled. (B) Lean mass and fat mass of 5-week-old *Lmod3^PB/PB^* males (black bar, *n*=10) and *Lmod3^+/+^* males (white bar, *n*=5) measured by EchoMRI. ** indicates *P*<0.01. (C) Forelimb grip strength of 4-week-old *Lmod3^PB/PB^* males (black bar, *n*=4) and *Lmod3^+/+^* males (white bar, *n*=5). (D-G) H&E staining of TA muscles from a 5-week-old *Lmod3^PB/PB^* male (F,G) and a wild-type male littermate control (D,E). Scale bar: 50 µm. Yellow arrows: atrophic muscle fibers with internalized nuclei.

Autopsy revealed that *Lmod3^PB/PB^* mice are much leaner than wild type (Fig. 2A). Quantitative EchoMRI analysis confirmed that lean mass, but not fat mass, were dramatically decreased in *Lmod3^PB/PB^* mice (Fig. 2B). The grip-strength assay showed that forelimb grip strength of *Lmod3^PB/PB^* mice was less than 50% of that of *Lmod3^+/+^* controls (Fig. 2C). These results indicate that *Lmod3* deficiency results in a skeletal muscle defect.

We further examined the *Lmod3^PB/PB^* muscle defect histologically in cross-sections of multiple muscles stained with hematoxylin and eosin (H&E), and observed atrophic myofibers in tibialis anterior (TA), gastrocnemius and quadriceps muscles of *Lmod3^PB/PB^* mice, but not controls (Fig. 2D-G and supplementary material Fig. S3A-F). In addition, internalized nuclei were detected in these small myofibers (Fig. 2G and supplementary material Fig. S3B,D,F). However, soleus muscles in *Lmod3^PB/PB^* mice appeared normal (supplementary material Fig. S3G,H).

Internalized myonuclei is one of the characteristics of regenerating fibers. However, the atrophic fibers with internal nuclei in the *Lmod3^PB/PB^* TA muscles were negative when stained with an embryonic myosin heavy chain antibody, a marker for active regenerating fibers (supplementary material Fig. S4A-E). Furthermore, typical histopathological features of degenerative muscles, including fiber necrosis and cellular infiltration, were not observed in the H&E staining of the *Lmod3^PB/PB^* muscles (Fig. 2F and supplementary material Fig. S3). These results suggest that the fibers with internal nuclei in the *Lmod3^PB/PB^* muscles are not undergoing degeneration or regeneration.

### Disorganized sarcomeric structure and nemaline bodies are present in *Lmod3^PB/PB^* muscles

**Fig. 3.**
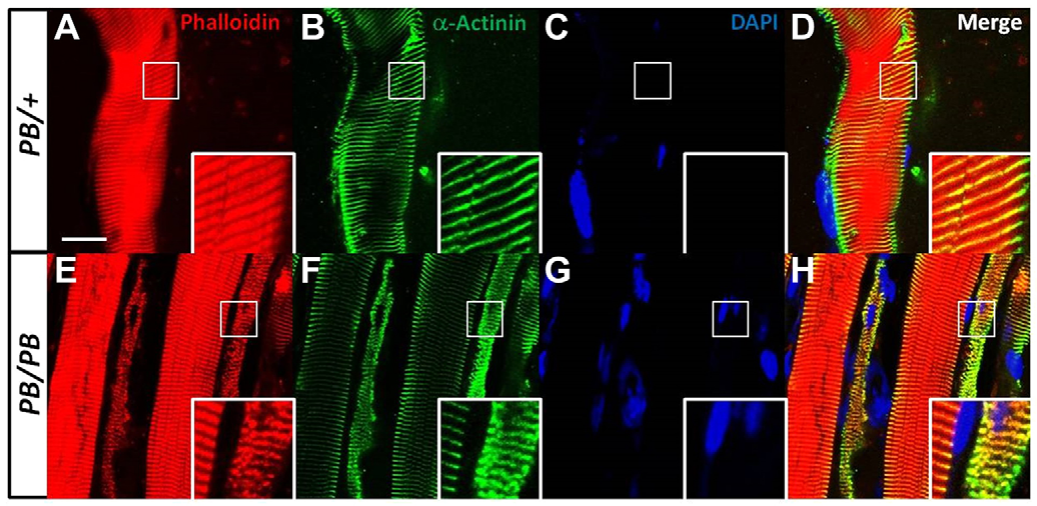
**Disorganized sarcomere structure in *Lmod3^PB/PB^* muscles.** Longitudinal sections of 4-week-old TA muscles from *Lmod3^PB/PB^* or *Lmod3^PB/+^* mice stained with phalloidin (A,E), α-actinin (B,F), DAPI (C,G) or merged (D,H). Scale bar: 20 µm. Inlets show magnification of marked regions.

To evaluate the sarcomeric structure in *Lmod3^PB/PB^* muscles, we examined the sarcomeres in the longitudinal sections of the TA muscles by simultaneous phalloidin staining for the F-actin and α-actinin antibody staining to label the Z-line. In comparison to the highly regular pattern in the *Lmod3^PB/+^* control (Fig. 3A), the phalloidin labeling in many of *Lmod3^PB/PB^* myofibers was narrower and highly disorganized (Fig. 3E). Consistent with the phalloidin staining result, the Z-lines were widened and disorganized in many of *Lmod3^PB/PB^* myofibers (Fig. 3F). In total, 62.6±2.2% of the *Lmod3^PB/PB^* TA myofibers displayed a disorganized sarcomere structure. Although, in many of the mutant myofibers, the sarcomere structure is too disorganized to measure the length of the thin filaments, we noted that the less disorganized ones are shorter than the control.

**Fig. 4.**
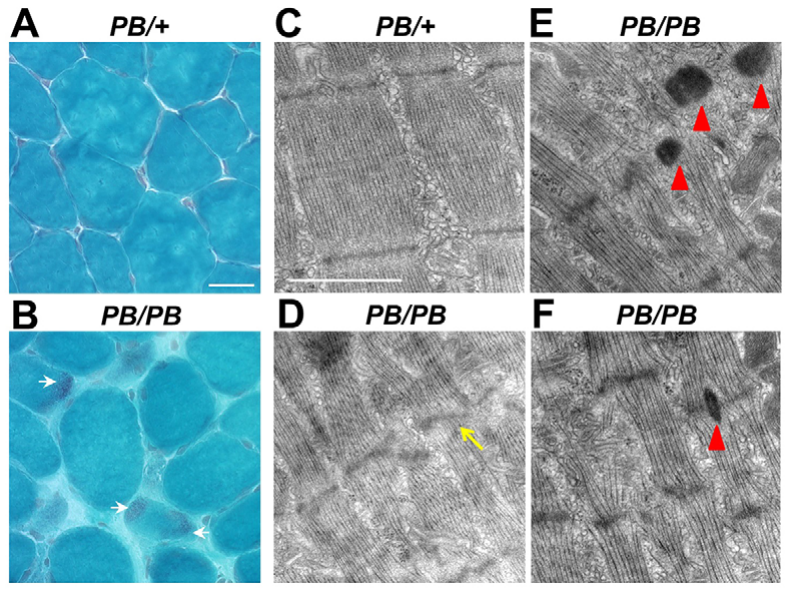
**Disorganized Z-line and nemaline bodies in *Lmod3^PB/PB^* muscles.** (A,B) Modified Gomori trichrome staining on 4-week-old TA muscles from *Lmod3^PB/+^* and *Lmod3^PB/PB^* mice. Scale bar: 50 µm. White arrows: nemaline bodies. (C-F) Electron microscopy images of EDL muscles from 6-week-old *Lmod3^PB/+^* (C) and *Lmod3^PB/PB^* (D-F) mice. Scale bar: 1 µm. Yellow arrow: Z-line streaming. Red arrowheads: nemaline bodies.

To visualize the nemaline bodies at the light microscopic level, we performed modified Gomori trichrome staining on multiple muscles. Nemaline bodies were present predominantly in the atrophic fibers as small dark dots in TA, quadriceps and gastrocnemius muscle in *Lmod3^PB/PB^* mice, but not controls (Fig. 4A,B and supplementary material Fig. S5A-C). Such signals were not found in soleus, a type-I-predominant muscle (supplementary material Fig. S5D).

To investigate the ultrastructure of the sarcomere, transmission electron microscopy was performed on extensor digitorum Longus (EDL) and TA muscles (Fig. 4C-F and supplementary material Fig. S6). The Z-lines in *Lmod3^PB/+^* sarcomeres were thin and well-organized (Fig. 4C and supplementary material Fig. S6A), whereas, in *Lmod3^PB/PB^* myofibrils, we found many widened Z-lines with local Z-line streaming (Fig. 4D and supplementary material Fig. S6B). More importantly, electron-dense nemaline bodies were present at the location of the Z-line in *Lmod3^PB/PB^* myofibrils (Fig. 4E-F and supplementary material Fig. S6C,D).

### Disruption of *Lmod3* causes atrophy that is specific to fast myofibers

**Fig. 5.**
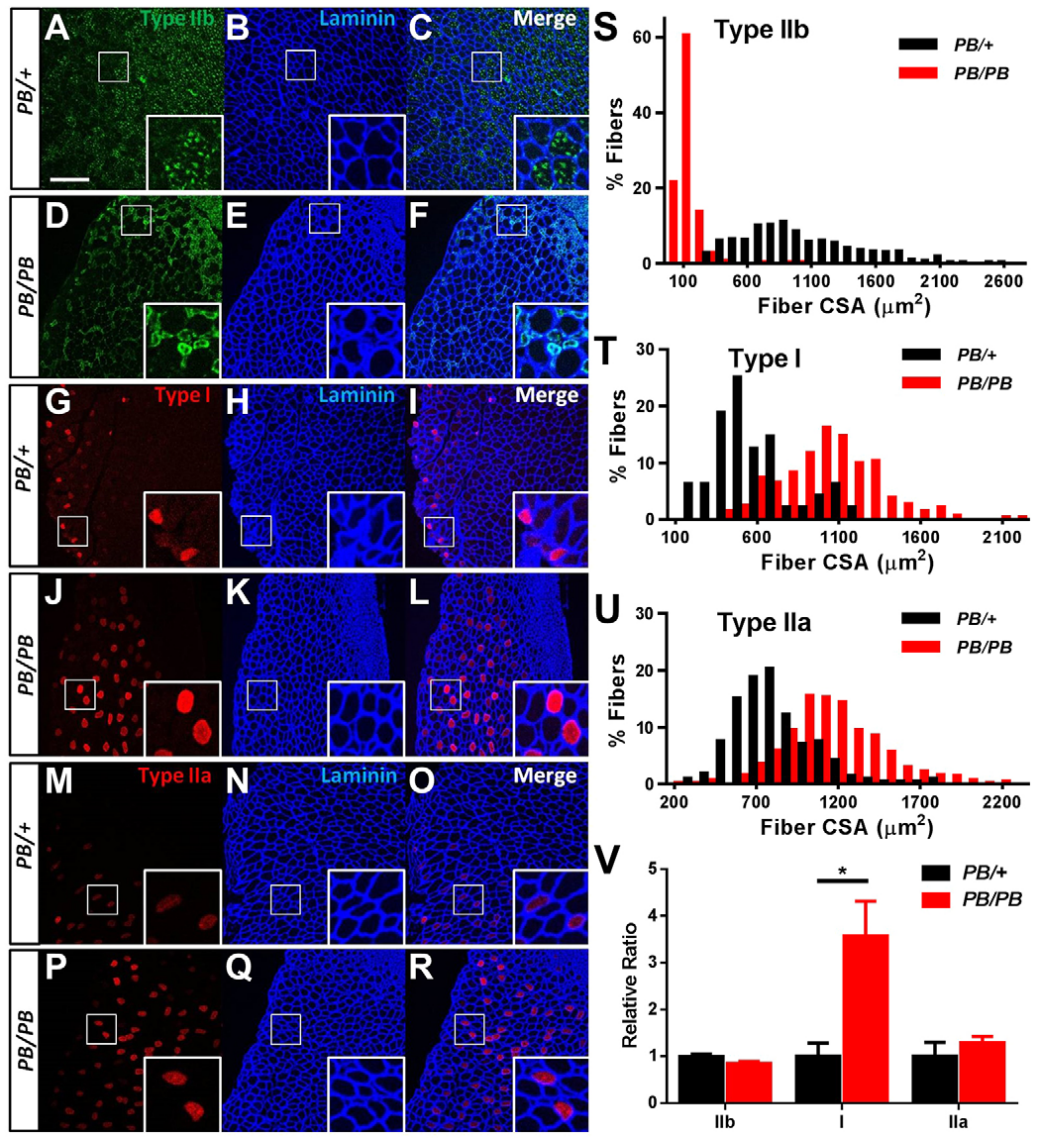
**Disruption of *Lmod3* causes atrophy specifically to fast myofibers.** (A-R) Cross-sections of 4-week-old TA muscles from *Lmod3^PB/+^* and *Lmod3^PB/PB^* mice stained with the antibodies indicated. Scale bar: 200 µm. (S-U) Size distribution of cross-sectional area (CSA) of type-IIb (S), type-I (T) and type-IIa (U) myofibers in TA muscles from 4-week-old *Lmod3^PB/+^* and *Lmod3^PB/PB^* mice (*n*=4). Panels S-U show *P*<0.001 in Kolmogorov–Smirnov test. (V) Relative ratio between the number of myofibers of a specific fiber type to the number of total myofibers in TA muscles from *Lmod3^PB/+^* and *Lmod3^PB/PB^* mice (*n*=4). *P<0.05.

Interestingly, in *Lmod3^PB/PB^* mice, only some of the myofibers were significantly smaller in size compared with controls (Fig. 2F,G and supplementary material Fig. S3). To examine whether the group of small myofibers in *Lmod3^PB/PB^* muscles belonged to a specific fiber type, the sections were stained with antibodies against different isoforms of myosin heavy chain. The sizes of type-IIb-positive (fast) fibers in *Lmod3^PB/PB^* muscles (TA, soleus and quadriceps) were much smaller than the *Lmod3^PB/+^* controls (Fig. 5A-F,S, supplementary material Fig. S7A,B,G and Fig. S8A,B,G). The sizes of type-I-positive (slow) and type-IIa-positive (intermediate) fibers in the mutant type-IIb-predominant muscles (TA and quadriceps) were larger than those in the controls (Fig. 5G-R,T-U and supplementary material Fig. S8H,I). However, in soleus, a type-I-predominant muscle, the sizes of type I fibers in the mutants were similar to those in the controls (supplementary material Fig. S7H) and type IIa fibers were slightly smaller (supplementary material Fig. S7I). Furthermore, there were more type I fibers in type-IIb-predominant muscles in *Lmod3^PB/PB^* mice than in the *Lmod3^PB/+^* controls (Fig. 5V and supplementary material Fig. S8J). Together, these data revealed that disruption of *Lmod3* results in atrophy specifically in fast myofibers.

In summary, *Lmod3^PB/PB^* mice recapitulate the muscular phenotypes shown in individuals with NM, including severe muscle weakness and the presence of nemaline bodies. Importantly, the *Lmod3*-deficient mice exhibit atrophy that is specific to fast myofibers, which makes it a unique animal model for NM and muscle atrophy.

## DISCUSSION

Recently, mutations in the *LMOD3* gene have been detected in a group of NM patients (Yuen et al., 2014). Subsequently, the expression of the *Lmod3* homologous gene was knocked-down in zebrafish and *Xenopus* by antisense morpholino (MO) (Nworu et al., 2015; Yuen et al., 2014). Both models display sarcomere disorganization and muscle dysfunction, which confirmed that Lmod3 plays an evolutionarily conserved role in muscle biology. In addition, the visibility of embryogenesis makes them useful models for studying Lmod3 function during embryonic myofibrillogenesis. However, neither of the models definitively shows the appearance of nemaline bodies, the hallmark of the NM disease. Furthermore, the relative short lifetime of MO RNA limits their potential for exploring Lmod3 function in adult muscles and in different fiber types. Therefore, a mammalian *Lmod3* mutant model will be a valuable resource for interrogating the underlying pathogenesis of NM and developing therapeutics.

Previously, we and others reported that the *piggyBac* (*PB*) transposon is able to transpose efficiently in the mouse germline and could be an efficient mutagen for insertional mutagenesis (Ding et al., 2005; Landrette et al., 2011; Ni et al., 2013; Rad et al., 2010). Here, we report a *PB* is inserted into the second intron of the *Lmod3* gene and almost abrogates *Lmod3* expression in homozygous mice. *Lmod3^PB/PB^* mice exhibit a series of NM-like phenotypes, including severe muscle weakness, muscle fiber atrophy, disorganization of the sarcomere structure and the presence of nemaline bodies, recapitulating the clinical presentations recently reported from the NM patients carrying mutations in the *LMOD3* gene (Yuen et al., 2014). Our results also indicate that *PB*-mediated germline mutagenesis in mice is a powerful genetic approach to discover human disease genes and generate new disease mouse models. During the revision of our manuscript, another *Lmod3* mutant mouse model with NM was generated by TALEN mutagenesis (Cenik et al., 2015). The phenotypes reported by Olsen and colleagues are similar to ours except that they also observed atrophy in soleus muscle. One possible explanation is a difference in genetic backgrounds, because the TALEN-induced mutant is in a C57BL/6×C3H mixed background, whereas ours is in a C57BL/6 background.

Several mouse models of NM have been developed either by knocking out (KO) an endogenous gene (e.g. Nebulin-KO, Klhl40-KO, Cfl2-KO) or overexpressing a mutant protein via transgenesis (Tg) or knock-in (KI) [e.g. Tg(ACTA1^D286G^), KI(ACTA1^H40Y^), Tg(TPM3^M9R^)] (Agrawal et al., 2012; Bang et al., 2006; Corbett et al., 2001; Garg et al., 2014; Ravenscroft et al., 2011a,b; Witt et al., 2006). Generally these mice display defects in sarcomere structure, muscle weakness and nemaline bodies, although with various age of onsets and severities. However, characterization of *Lmod3^PB/PB^* mice revealed specific atrophy of fast fibers. The phenotype has not been previously reported in other mouse models for NM or other congenital myopathy. In human skeletal muscles, LMOD3 is localized near the pointed end of the thin filaments (Yuen et al., 2014). However, it was observed that its location from the Z-disk is differentially far away in fast myofibers in comparison to slow myofibers, raising the possibility that LMOD3 might play different roles in the two fiber types (Yuen et al., 2014). Alternatively, differential expression of functionally redundant molecules in the fast and slow fibers could be the reason for the *Lmod3^PB/PB^* phenotype, because Tmod4 has been shown to be able to replace Lmod3 function in *Xenopus* (Nworu et al., 2015). Notably, atrophy of fast fibers and hypertrophy of slow fibers are also found in individuals with NM type 6 (NM6) (Olivé et al., 2010). The *KBTBD13* gene is mutated in individuals with NM6 and encodes a muscle-specific substrate adaptor of a ubiquitin ligase (Sambuughin et al., 2012). It is not clear whether sarcomere malfunction contributes to NM6 pathogenesis. Given the similar fiber-type-restricted phenotype, it is possible that sarcomere defects resulting from dysregulation of Lmod3 could be involved in the pathogenesis of NM6. Interestingly, preferential atrophy of fast fibers is a common feature of pathophysiological conditions, including cancer cachexia and sarcopenia, as well as glucocorticoid-induce myopathy (Lexell, 1995; Mendell and Engel, 1971; Schakman et al., 2013). Thus, *Lmod3^PB/PB^* mice offer a unique mammalian model for studying myopathy mechanisms and for developing therapeutics.

## MATERIALS AND METHODS

### Animals

The founder *Lmod3^PB/+^* mouse was generated by random germline transposition of *PB[Act-RFP]*, a *PB* transposon, on the C57BL/6J background (Jackson Laboratories, USA) (Ding et al., 2005). All animal experimental protocols used in this study were reviewed and approved by Yale Institutional Animal Care and Use Committee. The data presented are from male mice unless mentioned otherwise.

### Quantitative RT-PCR

Total RNA was isolated from TA muscles from 5-week-old mice by using TRIzol reagent (Invitrogen, USA) and used in iScript reaction (Bio-Rad, USA) to synthesize the cDNA. Quantitative RT-PCR with iTaq Universal SYBR Green Supermix (Bio-Rad, USA) was performed on StepOne system (Applied Biosystems, USA) to determine gene expression using the relative standard-curve method. β-actin was used as an internal control. Primers targeting *Lmod3* were: 5′-CAATGTCGCCTACCTTAAACCT-3′ and 5′-TGCTGTTCTAGGTGACTCTGCT-3′. Primers targeting β-actin were: 5′-GGCTGTATTCCCCTCCATCG-3′ and 5′-CCAGTTGGTAACAATG-CCATGT-3′.

### Western blotting

TA muscles were dissected and lysed in RIPA buffer with Complete Mini protease inhibitors (Roche, USA) at 4°C. Total protein was quantified by the BCA Assay (Pierce, USA). Protein samples were separated by SDS/PAGE according to standard western blotting procedures and transferred to nitrocellulose membranes (Bio-Rad, USA), followed by blocking with 5% skim milk for 1 h. Blots were incubated with primary antibodies to Lmod3 (1:1000; HPA036034; Sigma, USA), Lmod2 (1:400; AP10364a; Abgent, USA) or β-tubulin (1:4000; T5168; Sigma, USA). Anti-rabbit and anti-mouse IgG antibodies conjugated to horseradish peroxidase were used (1:5000; Jackson ImmunoResearch Laboratories, USA) for protein detection, and signal was visualized using enhanced chemiluminescence (Perkin-Elmer, USA).

### Body composition

Lean mass and fat mass were determined by EchoMRI-100 analyzer according to the manufacturer's instruction (EchoMRI LLC, USA).

### Grip strength

Forelimb grip strength was measured with a grip-strength meter according to the manufacturer's instruction (Columbus Instruments, USA).

### Histochemistry

Cross-sections (10 µm) of isopentane-frozen muscles were stained with H&E or modified Gomori trichrome with standard histochemical techniques (Sheehan and Hrapchak, 1987). Light microscopic images were captured using a Zeiss AxioPhot microscope with an AxioCam 105 color camera.

### Electron microscopy

The mice were cardiacally perfused with Karnovsky fixative solution. TA and EDL muscles were fixed in 3% glutaraldehyde in 0.1 M cacodylate buffer pH 7.4 for 60 min, washed 3×10 min in 0.1 M cacodylate buffer, post-fixed with 1% potassium ferrocyanide reduced OsO_4_ for 3 h on ice, dehydrated through graded methanol, and embedded in EMbed 812 resin. Ultrathin sections (60 nm) were contrasted with uranyl acetate and lead citrate, and viewed in a Hitachi H600 TEM.

### Immunofluorescence

Longitudinal or cross-sections (10 µm) of isopentane-frozen muscles were subjected to immunofluorescence assay according to standard methods. Primary antibodies were to: α-actinin (A7811, Sigma-Aldrich, USA), laminin (L9393, Sigma-Aldrich, USA), myosin heavy chain IIb (BF-F3, Developmental Studies Hybridoma Bank, USA), myosin heavy chain I (BA-D5, Developmental Studies Hybridoma Bank, USA), myosin heavy chain IIa (SC-71, Developmental Studies Hybridoma Bank, USA), embryonic myosin heavy chain (F1.652, Developmental Studies Hybridoma Bank, USA). Rhodamine phalloidin (R415, Invitrogen, USA) and Alexa-Fluor-405, -488 or -633 secondary antibodies (Invitrogen, USA) were used for immunofluorescence detection. Images were captured using a Leica TSC SP8 confocal laser scanning microscope with Leica Application Suite Advanced Fluorescence 4.0. Number or size of the muscle myofibers were quantified using the ImageJ program (National Institutes of Health, USA).

### Statistics

Data were presented as mean±s.e.m. For statistical analysis, two-tailed unpaired Student's *t*-tests were used unless otherwise stated. *P*<0.05 was considered significant. Prism 6 (GraphPad Software, USA) was used for plotting. Error bars represent s.e.m.

## Supplementary Material

Supplementary Material
