# Supplementary material for: Leiomodin-3-deficient mice display nemaline myopathy with fast-myofiber atrophy
Source: Dis Model Mech. 2015 Jun 1;8(6):635–41. doi: 10.1242/dmm.019430 (PMC4457035; doi:10.1242/dmm.019430)
Supplement: Supplementary Material [file supp_8_6_635__index.html]

Leiomodin-3-deficient mice display nemaline myopathy with fast-myofiber atrophy — Supplementary Material 

# Leiomodin-3-deficient mice display nemaline myopathy with fast-myofiber atrophy

## DMM019430 Supplementary Material

**Files in this Data Supplement:**

- **Supplementary Material**
