# Supplementary material for: Leiomodin-3-deficient mice display nemaline myopathy with fast-myofiber atrophy
Source: Dis Model Mech. 2015 Jun 1;8(6):635–41. doi: 10.1242/dmm.019430 (PMC4457035; doi:10.1242/dmm.019430)
Supplement: Supplementary Material [file supp_8.6.635_DMM019430.pdf]

Supplementary Materials

Figure S1. Lmod2 expression is comparable between *Lmod3*$^{PB/PB}$ mice and *Lmod3*$^{+/+}$ mice. Western blotting of 5-week-old mouse TA muscles stained with antibodies against Lmod2 and β-Tubulin (as loading control).

Figure S2. Disruption of Lmod3 expression causes growth retardation in *Lmod3*$^{PB/PB}$ female mice. Growth curves of female *Lmod3*$^{+/+}$ mice (n=6), *Lmod3*$^{PB/+}$ mice (n=13) and *Lmod3*$^{PB/PB}$ mice (n=11) are presented. ^, # or * indicates P <0.05; ^^, ## or ** indicates P <0.01; ### or *** indicates P <0.001.

Figure S3. Atrophic and internally nucleated fibers in *Lmod3*$^{PB/PB}$ muscles. Cross sections of different muscles from 4-week-old *Lmod3*$^{PB/+}$ and *Lmod3*$^{PB/PB}$ mice were stained by H&E. Scale bar: 50 µm. Yellow arrows: atrophic fibers with internalized nuclei.

Figure S4. Internally nucleated fibers in *Lmod3*$^{PB/PB}$ muscles are not regenerative. (**A-E**) Cross sections of 4-week-old TA muscles from *Lmod3*$^{PB/PB}$ mice stained with antibodies indicated. Scale bar: 200 µm. Yellow arrows: atrophic type IIb fibers with internalized nuclei. (**F**) Percentage of internal nucleated fibers in 4-week-old TA muscles from *Lmod3*$^{PB/+}$ and *Lmod3*$^{PB/PB}$ mice.

Figure S5. Modified Gomori trichrome staining on different muscles from *Lmod3*$^{PB/PB}$ mice. White arrow: nemaline bodies. Scale bar: 50 µm.

Figure S6. Disorganized Z-line and nemaline bodies in *Lmod3*$^{PB/PB}$ TA muscles. Electron microscopy images of TA muscles from 4-week-old *Lmod3*$^{PB/+}$ (**A**) and *Lmod3*$^{PB/PB}$ mice (**B-D**). Scale bar: 1 µm. Yellow arrow: Z-line streaming. Red arrowhead: nemaline body.

Figure S7. Disruption of *Lmod3* causes atrophy specific to fast myofibers in soleus muscle. (**A-F**) Cross sections of 4-week-old soleus muscles from *Lmod3*$^{PB/+}$ and *Lmod3*$^{PB/PB}$ mice stained with antibodies indicated. Scale bar: 200 µm. (**G-I**) Size distribution of CSA of type IIb (**G**), type I (**H**) and type IIa (**I**) myofibers in soleus muscles from 4-week-old *Lmod3*$^{PB/+}$ and *Lmod3*$^{PB/PB}$ mice. (**G**) and (**I**) show $P< 0.001$ in Kolmogorov–Smirnov test. (**V**) Relative ratio between number of myofibers of a specific fiber type to number of total myofibers in soleus muscles from *Lmod3*$^{PB/+}$ and *Lmod3*$^{PB/PB}$ mice (N=3).

Figure S8. Disruption of *Lmod3* causes atrophy specific to fast myofibers in quadriceps muscle. (**A-F**) Cross sections of 4-week-old quadriceps muscles from *Lmod3*$^{PB/+}$ and *Lmod3*$^{PB/PB}$ mice stained with antibodies indicated. Scale bar: 200 µm. (**G-I**) Size distribution of CSA of type IIb (**G**), type I (**H**) and type IIa (**I**) myofibers in quadriceps muscles from 4-week-old *Lmod3*$^{PB/+}$ and *Lmod3*$^{PB/PB}$ mice. (**G-I**) show $P< 0.001$ in Kolmogorov–Smirnov test. (**V**) Relative ratio between number of myofibers of a specific fiber type to number of total myofibers in quadriceps muscles from *Lmod3*$^{PB/+}$ and *Lmod3*$^{PB/PB}$ mice (N=3). *$P<0.05$.

Figure S1

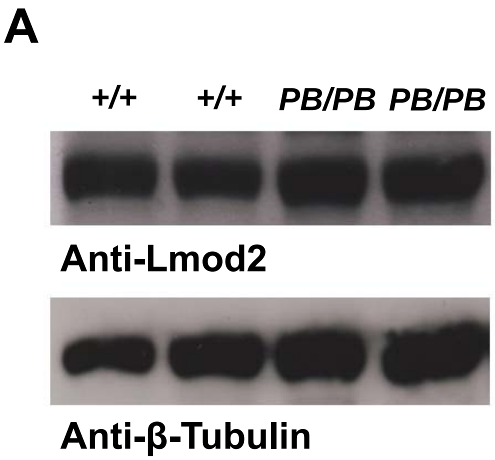

Figure S2

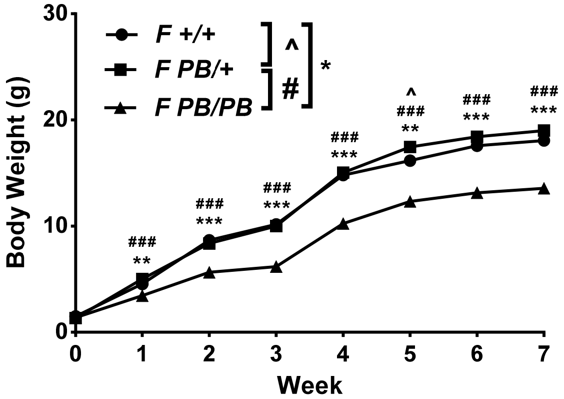

Figure S3

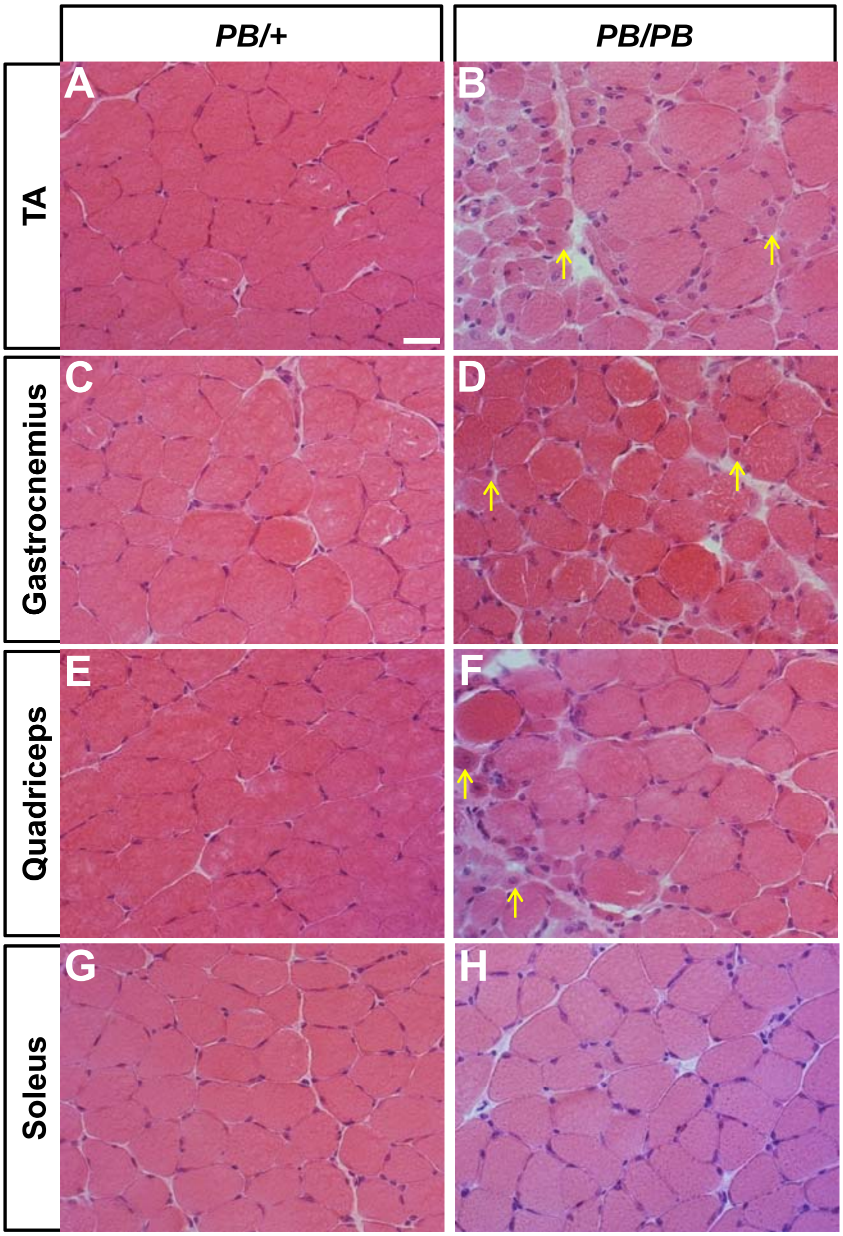

Figure S4

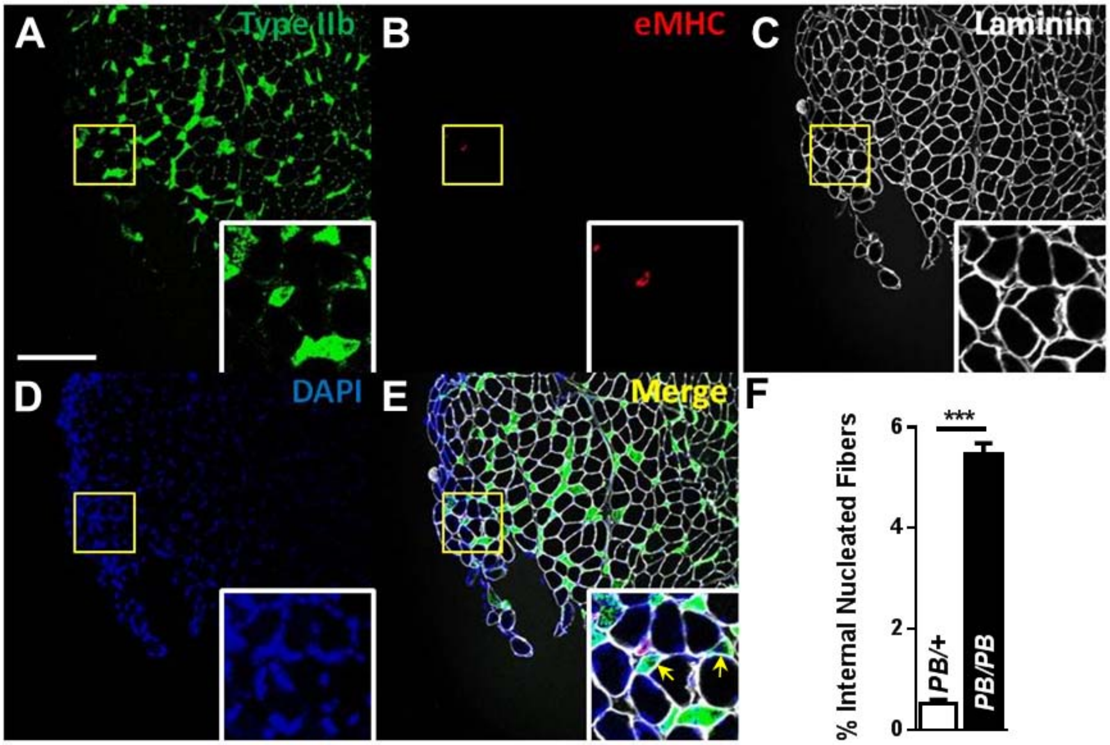

A
Type IIb
B
eMHC
C
Laminin
D
DAPI
E
Merge
F
% Internal Nucleated Fibers
0
2
4
6
***
PB/+
PB/PB

Figure S5

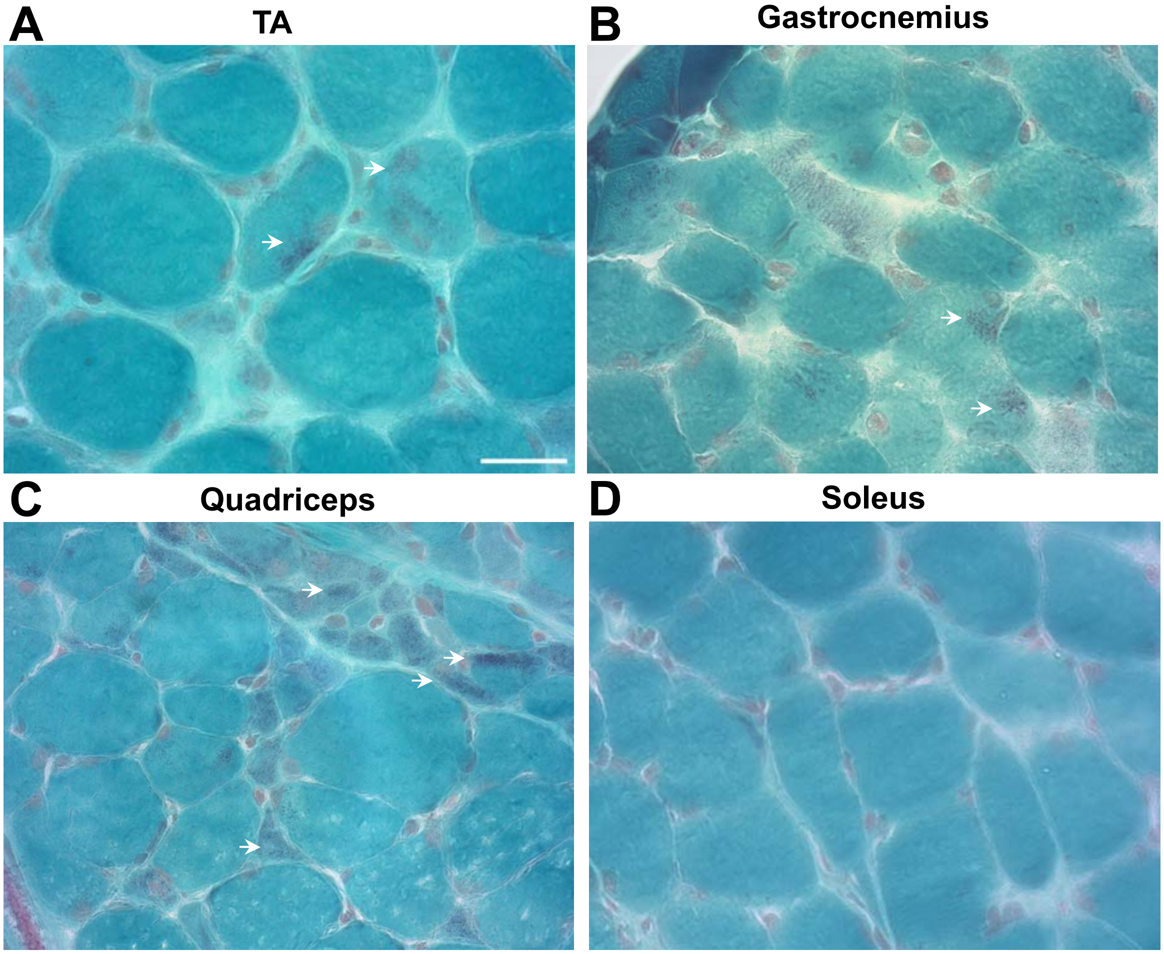

Figure S6

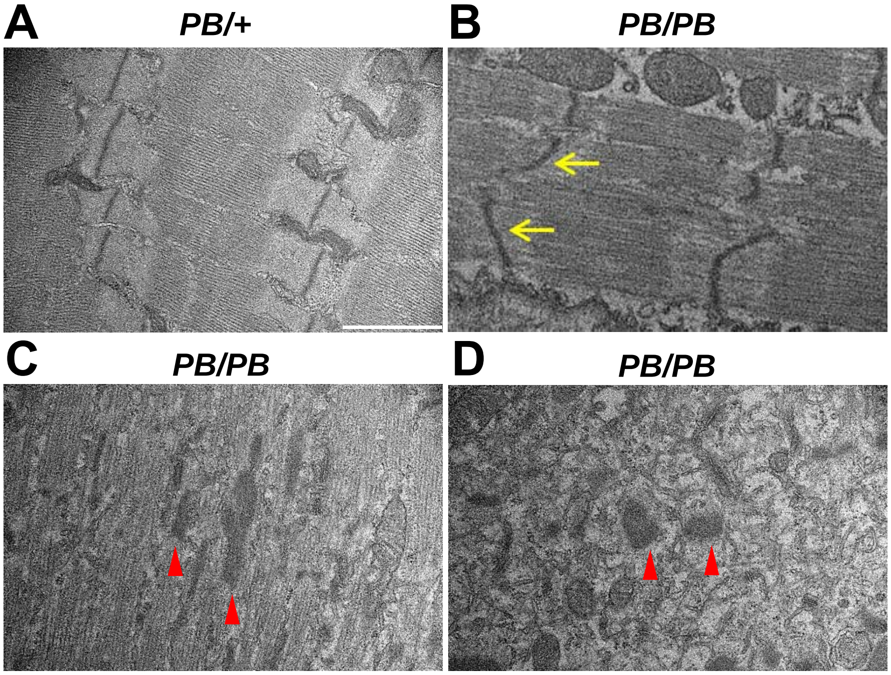

Figure S7

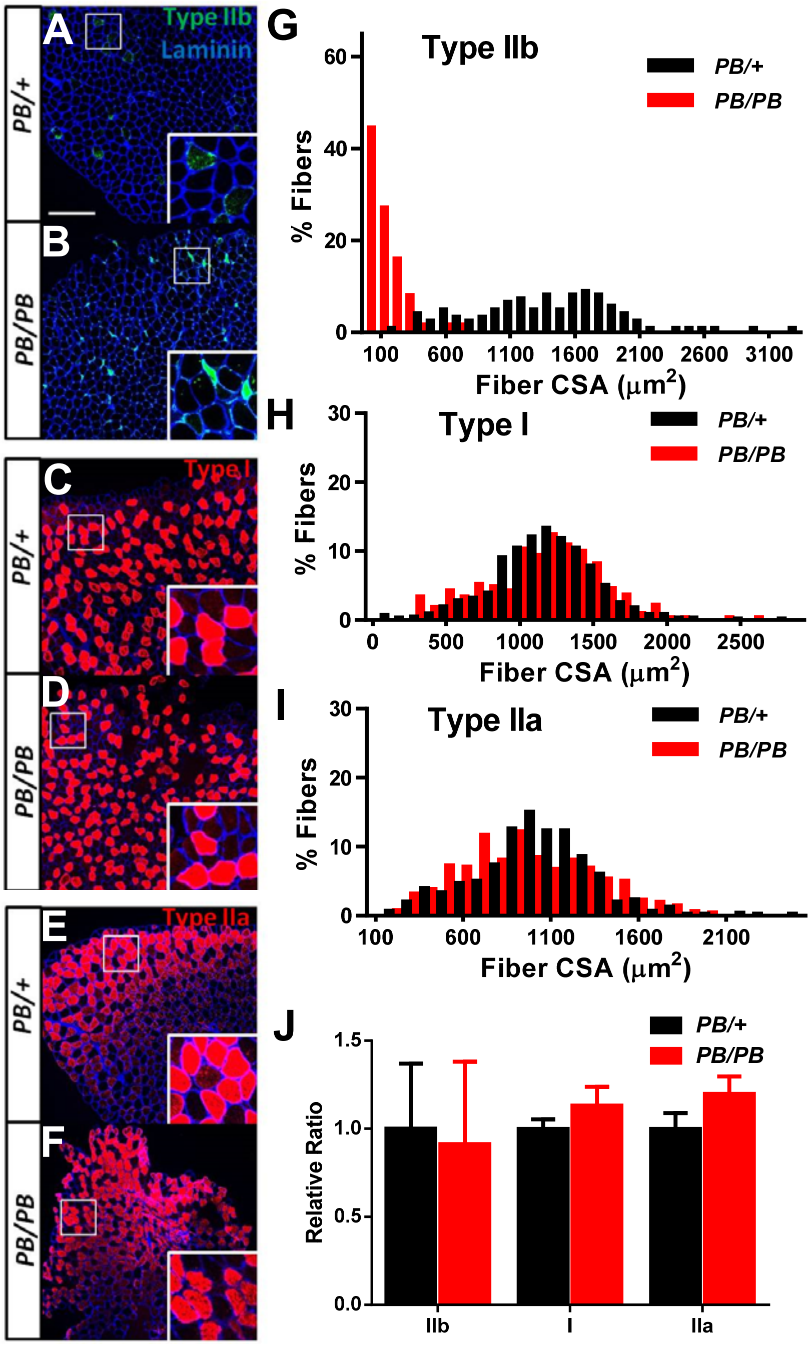

A
Type IIb
Laminin
PB/+
B
PB/PB
C
Type I
PB/+
D
PB/PB
E
Type IIa
PB/+
F
PB/PB
G
Type IIb
PB/+
PB/PB
% Fibers
60
40
20
0
100
600
1100
1600
2100
2600
3100
Fiber CSA (μm2)
H
Type I
PB/+
PB/PB
% Fibers
30
20
10
0
0
500
1000
1500
2000
2500
Fiber CSA (μm2)
I
Type IIa
PB/+
PB/PB
% Fibers
30
20
10
0
100
600
1100
1600
2100
Fiber CSA (μm2)
J
PB/+
PB/PB
Relative Ratio
1.5
1.0
0.5
0.0
IIb
I
IIa

Figure S8

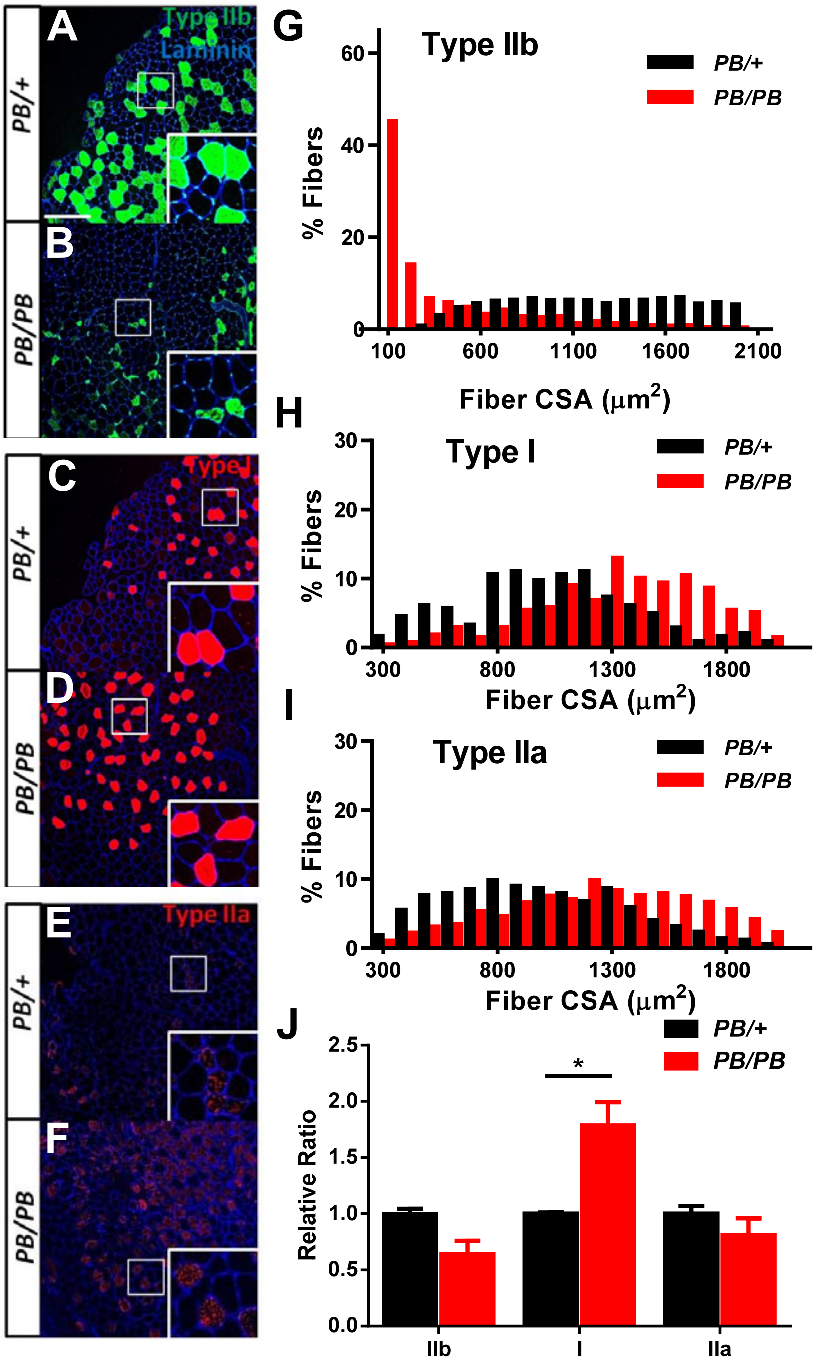

A
Type IIb
Laminin
PB/+
B
PB/PB
C
Type I
PB/+
D
PB/PB
E
Type IIa
PB/+
F
PB/PB
G
Type IIb
PB/+
PB/PB
% Fibers
60
40
20
0
100
600
1100
1600
2100
Fiber CSA (μm2)
H
Type I
PB/+
PB/PB
% Fibers
30
20
10
0
300
800
1300
1800
Fiber CSA (μm2)
I
Type IIa
PB/+
PB/PB
% Fibers
30
20
10
0
300
800
1300
1800
Fiber CSA (μm2)
J
PB/+
PB/PB
*
Relative Ratio
2.5
2.0
1.5
1.0
0.5
0.0
IIb
I
IIa
